# Sick leave among home-care personnel: a longitudinal study of risk factors

**DOI:** 10.1186/1471-2474-5-38

**Published:** 2004-11-08

**Authors:** Eva L Horneij, Irene B Jensen, Eva B Holmström, Charlotte Ekdahl

**Affiliations:** 1 Ramlösa Clinic, Ramlösa Brunnshotell, Helsingborg, Sweden; 2 Department of Physical Therapy, Lund University, Lund, Sweden; 3 Section for Personal Injury Prevention, Karolinska Institute, Stockholm, Sweden

## Abstract

**Background:**

Sick leave due to neck, shoulder and back disorders (NSBD) is higher among health-care workers, especially nursing aides/assistant nurses, compared with employees in other occupations. More information is needed about predictors of sick leave among health care workers. The aim of the study was to assess whether self-reported factors related to health, work and leisure time could predict: 1) future certified sick leave due to any cause, in nursing aides/assistant nurses (*Study group I*) and 2) future self-reported sick leave due to NSBD in nursing aides/assistant nurses (*Study group II*).

**Methods:**

*Study group *I, comprised 443 female nursing aides/assistant nurses, not on sick leave at baseline when a questionnaire was completed. Data on certified sick leave were collected after 18 months. *Study group II *comprised 274 of the women, who at baseline reported no sick leave during the preceding year due to NSBD and who participated at the 18 month follow-up. Data on sick leave due to NSBD were collected from the questionnaire at 18 months. The associations between future sick leave and factors related to health, work and leisure time were tested by logistic regression analyses.

**Results:**

Health-related factors such as previous low back disorders (OR: 1.89; 95% CI 1.20–2.97) and previous sick leave (OR 6.40; 95%CI 3.97–10.31), were associated with a higher risk of future sick leave due to any cause. Factors related to health, work and leisure time, i.e. previous low back disorders (OR: 4.45; 95% CI 1.27–15.77) previous sick leave, not due to NSBD (OR 3.30; 95%CI 1.33–8.17), high strain work (OR 2.34; 95%CI 1.05–5.23) and high perceived physical exertion in domestic work (OR 2.56; 95%CI 1.12–5.86) were associated with a higher risk of future sick leave due to NSBD. In the final analyses, previous low back disorders and previous sick leave remained significant in both study groups.

**Conclusion:**

The results suggest a focus on previous low back disorders and previous sick leave for the design of early prevention programmes aiming at reducing future sick leave due to any cause, as well as due to NSBD, among nursing aides/assistant nurses. A multifactorial approach may be of importance in the early prevention of sick leave due to NSBD.

## Background

Over the past century, sick leave, mainly attributed to musculoskeletal disorders (MSD), has increased in Sweden, especially among women [1]. In 2003, the proportion of women on sick leave was higher than in any previous year [1], indicating more individual suffering and also increased cost for the community. The prevalence of neck, shoulder and back pain is higher among health care workers, especially among nursing aides, compared with employees in other occupations [2-4]. In 2002, women working within the health-care sector in Sweden had the highest proportion of sick leave, mainly attributed to MSD [1]. The need for effective early prevention strategies is evident. However, if customised prevention programmes are to be possible, more information is needed about predictors of future sick leave among women working in the nursing sector.

Many studies have focused on the association between risk factors and the reporting of neck, shoulder and back pain [5-8]. However, back pain is a recurrent problem, which may or may not influence participation in daily activities and the ability to work [9,10]. It has been shown that predictors of perceived pain may not be the same as predictors of future sick leave [11]. Thus, when constructing intervention programmes, focusing on individuals at risk of future sick leave, predictors associated with perceived pain should be distinguished from those of future sick leave.

Studies have pointed to different factors in different populations to relate with future sick leave. Demographic factors such as age may be associated with sick leave mainly in individuals with chronic neck-or-back disorders [12]. In some studies this association was, however, not verified, which may be due to a "healthy worker effect" [11,13]. Medical factors, such as, for example, previous experience of back disorders, perceived health or prior sick leave predicted future sick leave [11,12,14-16]. Self-reported high physical load increased the risk of prolonged sick leave in populations already on sick leave due to back pain [17,18]. Perceived high work load in combination with low decision latitude, hereafter called high-strain work, is assumed to have negative consequences on health [19,20]. Low social support may be a risk factor for the development of back pain and also for future sick leave [19,20]. The association between high work load, low decision latitude and low social support and future sick leave is, however, unclear [14,19].

Mental problems, such as, for example, anxiety, also increased the risk of future sick leave among nurses [19]. Moreover, economic problems exacerbated the risk of prolonged sick leave among patients with low back disorders [21]. A strained economic situation also tends to lead to a decreased probability that woman suffering low back pain will seek medical attention [22].

The effects of physical exercising on future sick leave are not clear. Sedentary activities in leisure time were, however, associated with a higher prevalence of back symptoms and sick leave [23]. Eriksen et al. [16,24] found that regular physical activity, such as brisk walks, aerobics or other forms of exercise for 20 minutes or more at least once a week predicted fewer sick leaves after 3 and 15 months among nursing aides.

To summarise, a large number of factors, demographic, medical, physical, psychosocial, psychological as well as socioeconomic, have been documented as risk factors for future sick leave in different working populations [14].

It has been concluded that there is a lack of studies on neck-and-back disorders among women working within special groups as for example the health-care sector [14]. Because studies on home-care personnel, analysing risk factors associated with neck, shoulder and back disorders, have mostly been cross-sectional, it is not possible to determine causality.

It is of interest to analyse whether the above-mentioned factors associated with future sick leave in different populations, can also predict future sick leave among nursing aides/assistant nurses working within the home-care service.

Working as a nursing aide/assistant nurse within the home-care service is generally physically heavy work, requiring repeated transfers and lifts of patients. The physical load on the spine depends on several factors, for example, the weight of the patient, work place design, work organisation, work technique, work equipment, the cooperation of the patient etc [25]. Even transfers of a light and cooperative patient imply high spinal loads and a risk of causing low back disorders [26]. An appropriate work technique may decrease the biomechanical load on the spine. However, work technique differs among individuals and should thus be studied on an individual level [25]. In the present study, self-reported measures were analysed and physical load on the spine were not included. Self-reported working positions did not associate with prolonged sick leave in individuals with chronic back pain [14]. In longitudinal studies on nurses and nursing aides, frequent lifting or repositioning of patients did not predict future sick leave [11,16]. However, the perception of physical exertion at work was a risk indicator for low back symptoms among nursing aides working in geriatric care [6]. The perception of physical exertion correlated also with the onset of low-back pain among nursing aides [7]. The risk of seeking care was higher among the nursing personnel who perceived high physical exertion in domestic work [27]. The impact of perception of physical exertion on future sick leave among nursing aides/assistant nurses is, to our knowledge, not known.

The aim of the present study was to assess whether a selection of self-reported measures of factors related to health, work and leisure time could predict

1. future, certified sick leave after 18 months, due to any cause, in a group of working nursing aides/assistant nurses (*Study group I*).

2. future, self-reported sick leave after 18 months, due to neck, shoulder and/or back disorders, in a group of working nursing aides/assistant nurses (*Study group II*).

The aim was based on the hypothesis that factors related to the workplace as well as factors related to perceived health and to leisure time are associated with future sick leave in female nursing aides/assistant nurses.

It was also hypothesised that predictors of future sick leave due to neck, shoulder and/or back disorders are different from predictors of sick leave due to any cause.

## Methods

This study was one part of a larger project, aiming at preventing or reducing disorders of the neck, shoulder or back among female nursing aides/assistant nurses, working within the home-care services. The definition of the titles of home-care personnel is heterogeneous. In the present paper, nursing aides had little or no formal training, while the assistant nurses had undergone two – three years secondary education in nursing or had long experience of the work as a nursing aide and about one-year further education.

The study was approved by the Ethical Committee of the Faculty of Medicine, University of Lund, Sweden (LU 286-95). All participants gave their written consent before participation.

### Subjects

**Figure 1:**
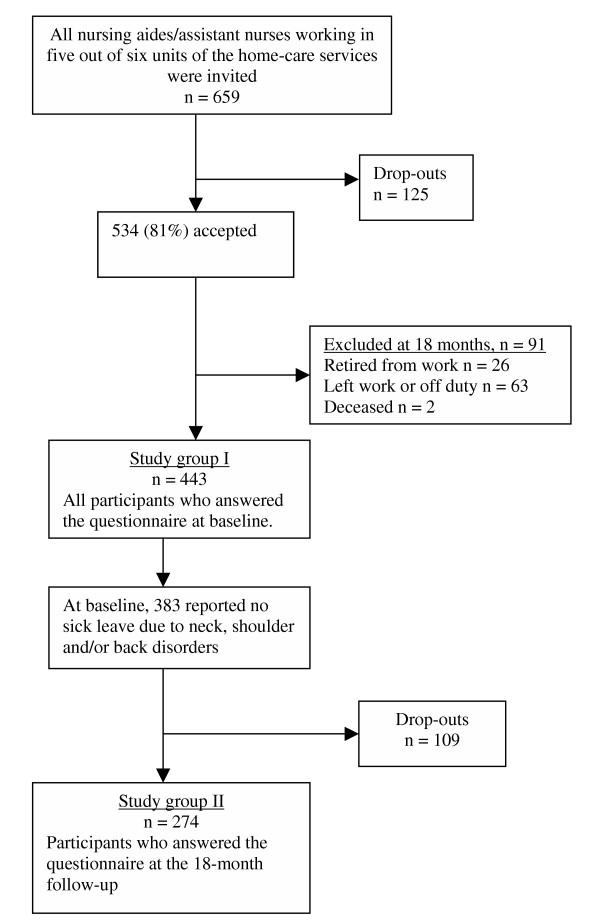
Study profile

The municipal home-care services were organised in six units situated in different geographically defined areas of a medium-sized town in the south of Sweden. Initially, all of the 659 women working in five of the six units were invited to participate in the prevention project. (One unit was excluded due to its participation in another study). The inclusion criteria were: Swedish speaking, permanently employed, not pregnant, in work and working at least 50% of full time. Participation was accepted by 534 (81%) of the women. The main reasons not to participate were: the opinion that the project was important only for younger staff, dissatisfaction with the work situation, participation in compulsory, further education for nursing aides to qualify as assistant nurse, lack of time or family reasons. As we wanted to elucidate predictors of future sick leave at an 18 month follow-up, women who at that time had retired from work, had left work or were off duty, were excluded from the analyses. Two women were deceased (n = 91). Thus, the final sample consisted of 443 nursing aides/assistant nurses (Figure 1).

At baseline, totally 282 of the originally 534 participants were randomized to one of two intervention groups, aiming at preventing neck, shoulder and back disorders (SM = Stress Management or IT = Individual Physical Training programmes) or to a Control group. Intervention groups and the Control group were evaluated and compared at the 18-month follow-up. No significant differences between the groups could be shown [39]. Thus, in the present study the groups on intervention programmes and the Control group were treated as a whole and called intervention.

#### Study group I

*Study group I *comprised 443 women who were not on sick leave when they completed the baseline questionnaire. Information on certified sick leave due to any cause during the six months preceding the 18-month follow-up was obtained from the National Social Insurance Board. The number of women who participated in intervention was 241 (54%). Demographic data, disorders, sick leave and participation in intervention for *Study group I *are presented in Table 1.

#### Study group II

*Study group II *comprised 274 women. At baseline, 383 women had reported no sick-leave due to symptoms from the neck, shoulder and/or back during the preceding 12 months. At the 18-month follow-up, 109 of these women did not fill in the questionnaire (Figure 1). The main known causes for dropping out at the 18-month follow-up were, not being able to fill in the questionnaires in time, mainly due to vacations, illnesses and refusal to take part, mainly due to lack of time. The reason for not participating was unknown for 69 of the women (18%). However, when further contacts were established with these women at a later stage, we were told that the reason for not responding was frequent reorganisations at work. Compared with participants, non-participants were, at baseline, more dissatisfied with social support at work (p = 0.03). No other differences between the participants and the drop-outs were shown. The number of women who participated in some intervention was 173 (64%). Demographic data, disorders, sick leave and participation in intervention for *Study group II *are presented in Table 1.

### Assessments

All participants were asked to fill in a questionnaire at the start of the study and after 6, 12 and 18 months. At baseline, the questionnaires were administered by the project nurse and filled in at the work place (*Study group I *and *Study group II*). At the 18-month follow-up, questionnaires were sent out to a contact person at each work place, who was made responsible for the distribution to the participants in the study. Each respondent received a stamped envelope in which she returned her own questionnaire to the project nurse (*Study group II*).

### Dependent variables

#### Sick leave due to any cause at the 18-month follow-up (Study group I)

Data about sick leave during the six months preceding the 18-month follow-up were obtained from the register of the National Social Insurance Board. In Sweden, the municipal authorities systematically record and report each day an employee is on sick leave to the National Social Insurance Board. Diagnoses are not reported to the Board. All days were counted as whole days irrespective of whether they were whole days or part of days. Due to skewed data, sick leave was dichotomized in 0 days/≥1 day (third quartile).

#### Sick leave due to disorders of the neck, shoulders and/or back at the 18-month follow-up (Study group II)

At all follow-ups the nursing aides/assistant nurses were asked: "Have you been on sick leave any time during the previous six months due to neck, shoulder and/or back disorders?". Response options were yes/no and constituted the dependent variable for *Study group II *at the 18-month follow-up. The validity of the responses was checked for all follow-up questionnaires against the general Nordic Musculoskeletal Questionnaire (NMQ) [28] i.e. the question concerning whether the disorder had been incapacitating during the preceding six months. A pattern was seen for women who reported sick leave due to neck, shoulder and/or back disorders at follow-ups by the fact that they reported in the NMQ that the disorders also had been incapacitating some time during the 6 months preceding the 18 month follow-up.

### Independent variables

#### Self-reported measures associated with health

##### Mental health

Anxiety and depression were assessed by the Hospital Anxiety and Depression Scale (HAD) [29] which consists of two subscales – one for anxiety and one for depression. Anxiety and depression were closely related (p = 0.000) and as depression levels are generally lower compared with anxiety levels in working populations [30], which was also the case in the present study, we selected anxiety as a measure of mental well-being. The subscale contains seven items ranging from 0 – 3, with higher scores reflecting greater anxiety. A sum of eight or more has been shown in comparisons with psychiatric interviews to reflect anxiety [29]. The Swedish version was tested and evaluated by Lundqvist et al. [31].

##### Musculoskeletal disorders

The prevalence of musculoskeletal disorders from the neck, shoulders and back was assessed by the general Nordic Musculoskeletal Questionnaire (NMQ) [28]. Participants were asked about pain, aches or discomfort some time during the preceding 12 months. The response options were yes/no.

##### Sick leave during the 12 months preceding baseline

Information on sick leave was obtained from the National Social Insurance Board.

Individuals in *Study group II*, who had been on sick leave, but who in the questionnaires had reported no sick leave due to symptoms from the neck, shoulder and/or back were assumed to be on sick leave due to other reasons. Work loss was recorded as whole working days. As all data on sick leave were extremely skewed, we chose to dichotomise the material. The third quartile was used as cut-off point, which for *Study group I *was 0 days/≥1 days and for *Study group II *<9 days/≥9 days.

#### Self-reported measures associated with work

##### Perceived physical exertion at work

The participants were asked: "What degree of physical exertion do you usually perceive in your present job?" [32]. The question was assessed according to Borg [33] and ranged from 6 (no exertion at all) to 20 (maximal exertion). High physical exertion at work was defined as 15 on the scale (corresponding "hard") or more (third quartile) [6].

##### Perceived work-related psychosocial factors

Psychosocial factors at work, such as social support, decision latitude and psychological load were assessed by a questionnaire developed by Rubenowitz [34,35]. The questionnaire considers five psychosocial factors: "Influence and control over work", "Supervisor climate", "Stimulus from the work itself", "Relation to fellow workers" and "Psychological load". Each factor comprises five items and each item has five fixed response alternatives from 1 to 5, where 1 means very unsatisfactory and 5 very satisfactory. A separate score, ranging from 1 to 5, is calculated on the mean of each factor. In the present analysis, "Supervisor climate" and "Relation to fellow workers" were defined as "Social support" and "Influence and control over work" and "Stimulus from the work itself" were defined as "Decision latitude" [35]. "Psychological load" together with "decision latitude" were defined as strain. High strain was equal to high psychological load in combination with low decision latitude [36]. The first quartile of the psychosocial factors were categorised as poor [34].

#### Self-reported measures associated with leisure time

##### Exercise and physical activity

The participants were asked: "To what extent have you performed physical activities or fitness training during the previous six months?" [32]. The question comprised eight options. A sedentary life style was assumed by the response "No exercise and very little physical activity" (score = 1).

##### Perceived physical exertion in domestic work

The participants were asked about physical exertion in domestic work in the same way as for physical exertion at work: "What degree of physical exertion do you usually perceive in your daily domestic work?" [32]. Response options ranged from 6 (no exertion at all) to 20 (maximal exertion) [33]. The cut-off point was 13 (third quartile) corresponding "somewhat hard".

##### Perceived psychological stress outside work

This question, originally developed for the Malmo Diet and Cancer study on 53000 Swedish men and women, was defined by "Have you lately felt mentally stressed or been under psychological pressure due to problems outside work?" Response options were yes/no [37].

##### Economic situation

Perceived satisfaction with her own economic situation was assessed by a seven-point scale where the first point represented "very bad" and the seventh point "Excellent, could not be better" [38].

### Statistical analysis

All logistic regression analyses were adjusted for age and intervention.

Three models were tested, namely, for factors related to health, to work and to leisure time. The associations between the independent variables and the outcome variables were first analysed for each of the three models by univariate logistic regression. Secondly, each model was analysed with all variables included in a backward stepwise multivariate regression analysis with a likelihood ratio test. Finally, a multivariate model including all the variables from the three models together was tested. The criteria for inclusion and exclusion were p = 0.05. The logistic regression analyses were tested for goodness of fit by means of the Hosmer and Lemeshow method. Factors were dichotomized by using cut-off points described in the literature. When no such literature was found, cut-off points were taken as the first or the third quartile for frequencies for *Study group II*. There were minimal differences between the quartiles of the two study groups. Thus, for the purpose of comparing the two groups, the same cut-off points were used. However, for sick-leave before baseline we chose two different cut-off points, as the third quartile differed between the two study groups, being 0 days for *Study group I *and 9 days for *Study group II*.

The sample size may vary in the different analyses due to missing values. Comparisons between drop-outs and participants were analysed by t-test or the chi-square test. Correlations between the exposure variables were calculated with Pearson correlation coefficients (r). All statistical calculations were performed using the SPSS 11.5.1 Software for Windows (SPSS Inc., Chicago, IL, USA).

## Results

In the univariate logistic regression analysis, there were minimal differences between odds ratios adjusted and not adjusted for age and intervention. Thus, in Tables 2,3,4,5, only adjusted odds ratios are presented.

### Predictors of future sick leave due to any cause (Study group I)

Age or participation in intervention (presented under section Methods-Subjects) did not have any effect on future sick leave due to any cause.

#### Self-reported factors related to health

Perceived disorders of the low back and sick leave during the 12 months preceding baseline predicted future sick leave due to any cause in the unadjusted and the adjusted univariate analyses as well as in the multivariate analysis (Table 2 and 5). Perceived disorders of the neck or shoulders did not predict future sick leave. Neck, shoulder and back disorders at baseline correlated with each other. Of the women who reported neck disorders, 88% also reported shoulder disorders (r = 0.57) and 75% reported low back disorders (r = 0.33). Of the women who reported shoulder disorders 71% reported low back disorders (r = 0.28).

#### Self-reported factors related to work

Factors related to work did not predict future sick leave due to any cause (Table 3 and 5).

#### Self-reported factors related to leisure time

Factors related to leisure time did not predict future sick leave in the univariate or in the multivariate analysis (Tables 4 and 5).

#### Final model Study group I

When all variables from the three models were entered into the final model, disorders of the low back during the preceding 12 months (OR: 1.95; CI: 1.23–3.10) and sick leave during the 12 months preceding baseline (OR: 6.61; CI: 4.08–10.73) were the only factors predicting sick leave due to any cause after 18 months.

### Predictors of future sick leave due to disorders of the neck, shoulders and/or back (Study group II)

A tendency towards a reduced risk of future sick leave due to neck, shoulder and/or back disorders was found for the women who participated in some intervention (OR: 0.46; CI: 0.21–1.01). Age did not have an impact on sick leave (OR: 1.02; CI: 0.98–1.06).

#### Self-reported factors related to health

Anxiety, low back disorders during the 12 months preceding baseline as well as sick leave due to reasons other than disorders of the neck, shoulders and/or back were significant predictors of sick leave after 18 months due to disorders of the neck, shoulders and/or back (p < 0.05) before and after adjustment for age and intervention (Table 2). When all characteristics related to health were entered into the multiple logistic regression model, disorders of the low back and sick leave due to reasons other than disorders of the neck, shoulders and/or back remained significant (Table 5). Neck disorders at baseline, correlated with shoulder disorders (r = 0.57) and with back disorders (r = 0.36). Of the nursing aides/assistant nurses who reported neck disorders, 87% also reported disorders of the shoulders and 75% reported disorders of the low back. Of those who reported disorders of the shoulders at baseline, 70% also reported disorders of the low back (r = 0.29).

#### Self-reported factors related to work

Before adjustment for age and intervention, high psychological load and high-strain work predicted future sick leave due to neck, shoulder and/or back disorders (OR: 2.28; CI:1.04–5.03 and OR: 2.42; CI: 1.09–5.37). However, after adjustments, only high-strain work demonstrated significant association with future sick leave (Table 3).

Two multivariate regression analyses were performed: one by entering psychological load and decision latitude separately into the model and one by analysing the same factors together (called strain). Only high-strain work predicted future sick leave due to disorders in the neck, shoulder and/or back (Table 5).

#### Self-reported factors related to leisure time

High perceived physical exertion in domestic work and perceived psychological stress outside work were associated with future sick leave due to neck, shoulder and/or back disorders both before and after adjustments for age and intervention (Table 4). In the multivariate model, high perceived physical exertion in domestic work remained significant predictor of future sick leave (Table 5).

#### Final model, Study group II

In the final model, where all variables from the three models were entered into the logistic regression analysis, perceived low back disorders some time during the 12 months preceding baseline and sick leave of > 9 days in the year before baseline remained significant predictors of sick leave due to neck, shoulder and/or back disorders at 18 months after baseline (OR: 7.36; CI: 1.67–32.43 and OR: 2.84; CI: 1.13–7.11 respectively).

## Discussion

The results of the present study indicated that only factors related to health were associated with future sick leave due to any cause in a group of nursing aides/assistant nurses. On the other hand, factors related to health, the workplace as well as leisure time were associated with future sick leave due to neck, shoulder and/or back disorders, indicating a multifactorial background for these disorders. Low back disorders and sick leave during the 12 months preceding baseline, were significantly associated with future sick leave in both *Study group I *and *Study group II*. Thus, the hypothesis that risk factors for future sick leave due to disorders in the neck, shoulders and/or back are different from sick leave due to any cause was only partly demonstrated. This is in concordance with other studies, which have also documented earlier experience of back pain and sick leave preceding baseline as predictors of future sick leave due to low back pain among nursing personnel [11] as well as in general populations [12,40]. Natvig et al. [15], showed that low back pain as a part of widespread pain predicted long-term disability due to any cause, while local low back pain did not. In the present study perceived disorders of the neck, shoulder and back correlated with each other, indicating wide spread disorders. Further, only disorders of the back and not of the neck or of the shoulders predicted sick leave in *Study group I *as well as in *Study group II*. One explanation for this finding may be the content of the work performed by a nursing aide/assistant nurse, with repeated repositioning, transfers and lifts of patients, which result in high spinal loads mainly on the low back [25,26]. Thus, it may be more difficult to perform this work with a vulnerable low back in comparison with vulnerable neck/shoulders. In the final analyses of *Study group I *and *Study group II*, including all variables from the three models, only low back disorders and previous sick leave remained significant. However, in *Study group II*, due to the small number of women on sick leave due to neck, shoulder and/or back disorders at the 18-month follow-up, results of this final analysis should be interpreted with caution.

Generally, most studies on predictors of future sick leave are made on men and few studies focusing on nursing personnel have been published. Thus, comparisons of the results from the current study with similar studies are limited.

The present study was performed during a period with frequent reorganisations and reductions of staff within the home-care services in the city studied, as in general in Sweden. A greater amount of work was performed faster than before and the category of patients, for whom the community home-care organisation took responsibility for, were more handicapped than previously. Thus, the physical and psychological stresses were generally exacerbated during the period studied. In the present study, among the factors related to work, only high-strain work (high psychological load in combination with low decision latitude) predicted future sick leave due to neck, shoulder and/or back. Marras et al. [41] showed that lifting combined with psychosocial stress, increases the load on the spine indicating an increased risk of back pain when working in physically demanding jobs in combination with high strain. Bourbonnais and Mondor [19] found that high-strain work reinforced the risk of future, short-term sick leave due to any cause among nurses. The inclusion of strain-reducing strategies in stress-management programmes for nursing aides/assistant nurses may, thus, decrease the risk of future sick leave due to back disorders, but should be further studied.

Women still do more unpaid work than men. In a 23-year perspective study, the physical work load decreased among men but not among women [42]. In the present study, we found that high physical exertion in domestic work predicted sick leave due to neck, shoulder and/or back disorders. Josephson et al. [27] found that the tendency to seek care was greater among nursing personnel who perceived that they did far too much domestic work. These findings point to the importance of also including factors outside work, even when customising early prevention programmes at the work place with the aim to reduce future sick leave due to neck, shoulder and back disorders among female nursing aides/assistant nurses.

We could not verify inactivity to be a predictor of future sick leave. The cut-off point was based on the assumption that a sedentary life style would predict future sick leave as shown in the study by Hildebrandt et al. [23]. In the study by Eriksen et al. [24], brisk walks, aerobics or gymnastics and other physical leisure-time activities for 20 minutes or more at least once a week protected against sick leave of more than 14 days, independent of diagnosis, after a 15-month follow-up period among nursing aides. The work as a nursing aide/assistant nurse is physically active, involving a great deal of walking. Few women reported a sedentary life at baseline. Thus, inactivity may not have been an appropriate cut-off point.

Moreover, in the present study, social support did not predict future sick leave. Since there was a significant initial difference in perceived social support between participants and non-participants in *Study group II*, the conclusions of this analysis are limited. However, neither in the *Study group I*, was social support related to sick leave. This finding is contradictory to the results of the study by Eriksen et al. on 5563 Norwegian nursing aides [16]. These authors found that social support was the most important work-related predictor of future sick leave due to any cause. Bourbonnais and Mondor [19] could also state a relationship between low social support and future sick leave among Canadian nurses. However, after adjustment for job strain, this relationship was no longer significant.

### Methodological considerations

Data on the dependent variable sick leave, for *Study group I *were obtained from the National Social Insurance Board and for *Study group II *were taken from the questionnaires. Systematically recorded, certified sick leave is assumed to be more valid than data on sick leave based on questionnaires [43]. For *Study group II*, questionnaires were filled in at the 6, 12 and 18 month follow-ups. In order to strengthen the validity of the question "Have you been on sick leave any time during the preceding six months due to neck, shoulder and/or back disorders", answers at the follow-ups were checked against the NMQ [28]. Participants who at baseline reported no sick leave due to neck, shoulder and/or back disorders could nevertheless, in the NMQ, indicate perceived disorders from the same regions but that these disorders had not been incapacitating. However, a pattern was seen for women who reported sick leave due to neck, shoulder and/or back disorders at follow-ups by the fact that they also, in the NMQ, reported that these disorders had been incapacitating. It may also be assumed that the recall bias was reduced and the sensitivity of the question about sick leave enhanced, as we did not ask the respondent to specify the number either of sick-days or of sick-leave episodes [43].

Age was not related to any reason for sick leave, which is in contrast with the results of a review study by Turner et al. [12] on people working in various occupations. They found an increased risk of sick leave with older age. However, the results of the present study are in concordance with other studies on home-care personnel and future sick leave [11,13], indicating a healthy worker effect among nursing aides/assistant nurses, which in turn may result in an underestimation of risk factors for future sick leave.

In *Study group II*, a large number of participants dropped out at the 18-month follow-up, which constitutes a risk of selection bias. The number of drop-outs is comparable with that found in other studies on nursing personnel [19,30,44]. In addition, except for social support at work (discussed earlier), participants did not differ from non-participants on any of the dependent variables. Thus, we regard the 274 participants in *Study group II *to be representative as a working group of nursing aides/assistant nurses.

The feasibility to generalise the results from *Study group I *to other women working as nursing aides/nursing assistants should be satisfactory, as all women participating at baseline were analysed at18 months and the data of the dependent variable, certified sick leave, were provided by the National Social Insurance Board.

In *Study group I*, 54% and in *Study group II*, 64% of the nursing aides/assistant nurses had participated in some intervention. The main purpose of this intervention project was not to reduce sick leave. The participants were informed that the aim of the project was to prevent or reduce pain and discomfort of the neck, shoulder and back. Sick leave was not mentioned. Thus, in our opinion, participation in intervention groups should not have biased the reporting of sick leave due to neck, shoulder and back disorders.

## Conclusions

The present study indicated that previous disorders of the low back and previous sick leave were the strongest predictors of future sick leave due to any cause as well as of future sick leave due to neck, shoulder and/or back disorders in a group of nursing aides/assistant nurses, who at baseline were at work. Previous neck or shoulder disorders did not predict future sick leave.

Moreover, factors related to health, work and leisure time were all related to sick leave due to neck, shoulder and/or back disorders, while factors related only to health predicted sick leave due to any cause.

The results point to the importance of a primary focus on previous low back disorders and previous sick leave when designing early prevention programmes for future sick leave among this working population. The results might also point to the importance of a multifactorial approach when customising early prevention programmes with the purpose to decrease future sick leave due to neck, shoulders and/or back disorders in a group of women working as nursing aides/assistant nurses.

## Competing interests

The author(s) declare that they have no competing interests.

## Authors' contributions

EHj participated in the design of the study, participated in collecting the data, performed the statistical analyses, and drafted the manuscript. IJ and EHm participated in the design of the study and in the progress and revision of the manuscript. CE participated in the progress and revision of the manuscript. All authors read and approved the final manuscript.

## Pre-publication history

The pre-publication history for this paper can be accessed here:



## Figures and Tables

**Table 1:** Demographic data, sick leave, proportions of symptoms from the neck, shoulder and low back during the 12 months preceding baseline, and proportions of women participating in some intervention.

	**Study group I^a ^** **n = 443**	**Study group II^b ^** **n = 274**
***Age, mean (SD)***	45 years (± 10)	45 years (± 10)
***Symptoms some time during the 12 months before baseline***		
*Neck*	59%	57%
*Shoulders*	66%	65%
*Low back*	62%	60%
***Number of days on sick leave due to any cause 12 months before baseline/6 months before 18-month follow-up, mean (SD)***	14 (38)/ 12 (31)	
***Numbers of days on sick leave 12 months before baseline (other reasons than disorders of the neck, shoulder and/or back)/ due to any cause 6 months before 18-month follow-up, mean (SD)***		10 (30)/ 9 (30)
***Duration of employment***		
0 – 4 *years*	3%	3%
5 – 10 *years*	22%	20%
11 – 20 *year*	47%	43%
>20 *years*	28%	34%
***Proportion of full-time work, mean (SD)***	81% (± 17)	81% (± 17)
***N:o of persons in household (including the person involved), mean (SD)***	2,7 (± 1.3)	2,8 (± 1.3)
***Living alone***	17%	16%
***Participation in intervention***		
*Stress Management group*	18%	21%
*Individual Physical Training group*	16%	18%
*Control group*	20%	25%

^a ^All participants at baseline. ^b ^Participants who at baseline reported no sick leave due to neck, shoulder and/or back disorders during the preceding 12 months and who answered the questionnaire at the 18-month follow-up.

**Table 2:** Self-reported factors related to health and associations with sick leave after 18 months due to any cause (*Study group I*)^a ^and due to neck, shoulder and/or back disorders (*Study group II*)^b^.

	***Number observed***	***Number observed***	***Odds Ratio***	***Odds Ratio***	***P value***	***P value***
***Variable***	***Study gp I^a ^(n = 443) *** ***N (cases)***	***Study gp II^b ^(n = 274)*** *** N (cases)***	***Study gp I OR *** ***(95%CI)***	***Study gp II OR (95%CI)***	***Study gp I***	***Study gp II***
Anxiety [0^c ^-21]						
- <8	332 (164)	217 (16)				
- ≥8	102 (55)	55 (11)	1.18 (0.76–1.85)	3.04 (1.31–7.06)	0.462	0.010
Neck disorders during the year before baseline						
- No	175 (79)	113 (10)				
- Yes	254 (137)	152 (16)	1.39 (0.94–2.06)	1.10 (0.47–2.55)	0.096	0.830
Shoulder disorders during the year before baseline						
- No	146 (67)	96 (10)				
- Yes	281 (147)	172 (16)	1.29 (0.86–1.93)	0.79 (0.34–1.85)	0.214	0.592
Low back disorders during the year before baseline						
- No	164 (64)	107 (4)				
- Yes	265 (152)	160 (22)	2.09 (1.40-3.12)	3.79 (1.26–11.44)	<10^-3^	0.018
Study group I						
Any sick leave during the year before baseline						
- 0 days	162 (35)					
- ≥1 days	281 (189)		7.67 (4.86–12.12)		<10^-3^	
Study group II						
Sick leave during the year before baseline (other reasons than disorders of the neck, shoulder and/or back)						
- <9 days		204 (16)				
- ≥9 days		70 (12)		2.64 (1.12–6.0)		0.021

^a ^All participants at baseline. ^b ^Participants who at baseline reported no sick leave due to neck, shoulder and/or back disorders during the preceding 12 months and who also answered the questionnaire at the 18-month follow-up. Minimum and maximum values are presented within square brackets. ^c ^Best possible value. CI = 95% confidence interval. Odds Ratios are adjusted for age and intervention

**Table 3:** Self-reported factors related to work and associations with sick leave after 18 months due to any cause (*Study group I*)^a ^and due to neck, shoulder and/or back disorders (*Study group II*)^b^.

	***Number observed***	***Number observed***	***Odds Ratio***	***Odds Ratio***	***P value***	***P value***
***Variable***	***Study gp I^a ^*** ***(n = 443) N (cases)***	***Study gp II^b ^*** ***(n = 274) N (cases)***	***Study gp I OR *** ***(95%CI)***	***Study gp II OR *** ***(95%CI)***	***Study gp I***	***Study gp II***
Perceived physical exertion at work [6^c ^-20]						
- <15	212 (102)	134 (12)				
- ≥15	229 (121)	140 (16)	1.21 (0.83–1.76)	1.33 (0.60–2.94)	0.318	0.487
Social support [1^d^-10]						
- >7.2	329 (168)	69 (9)				
- ≤7.2]	114 (56)	201(19)	0.94 (0.61–1.44)	0.77 (0.32–1.81)	0.770	0.54
Decision latitude [1^d^-10]						
- >6.2	311 (159)	86 (12)				
- ≤6.2	132 (65)	185 (16)	0.97 (0.64–1.46)	0.61 (0.27–1.37)	0.873	0.234
Psychological load [1^d^-5]						
- >2.6	303 (145)	183 (14)				
- ≤2.6	140 (79)	88 (14)	1.44 (0.96–2.17)	2.15 (0.97–4.77)	0.077	0.060
Strain (Dec^n^-Lat^de ^+ Psych load) [1^d^-15]						
- >8.8	321 (160)	194 (15)				
- ≤8.8	122 (64)	77 (13)	1.16 (0.76–1.76)	2.35 (1.05–5.26)	0.500	0.037

^a ^All participants at baseline. ^b ^Participants who at baseline reported no sick leave due to neck, shoulder and/or back disorders during the preceding 12 months and who also answered the questionnaire at the 18-month follow-up. Minimum and maximum values are presented within square brackets. ^c ^Best possible value. ^d ^Worst possible value. CI = 95% confidence interval. Odds Ratios are adjusted for age and intervention

**Table 4:** Self-reported factors related to leisure time and associations with sick leave after 18 months due to any cause (*Study group I*)^a ^and due to neck, shoulder and/or back disorders (*Study group II*)^b^.

	***Number observed***	***Number observed***	***Odds Ratio***	***Odds Ratio***	***P value***	***P value***
***Variable***	***Study gp I^a ^*** ***(n = 443) *** ***N (cases)***	***Study gp II^b ^*** ***(n = 274)*** *** N (cases)***	***Study gp I OR *** ***(95%CI)***	***Study gp II OR (95%CI)***	***Study gp I***	***Study gp II***
Physical activity and exercise						
- Physical activity and/or at least some exercise	422 (215)	261 (27)				
- No exercise and very little physical activity	17 (7)	12 (1)	0.67 (0.25–1.79)	0.77 (0.09–6.28)	0.418	0.805
Perceived physical exertion in domestic work [6^c ^-20]						
- <13	285 (147)	180 (13)				
- ≥13	156 (77)	88 (15)	0.96 (0.65–1.44)	2.36 (1.05–5.31)	0.855	0.038
Perceived psychological stress outside work						
- No	305 (160)	185 (13)				
- Yes	137 (63)	88 (14)	0.79 (0.53–1.19)	2.40 (1.06–5.43)	0.253	0.035
Satisfaction with economy [1^d^-7]						
- >4	282 (138)	179 (19)				
- ≤4	157 (84)	93 (8)	1.29 (0.86–1.93)	0.77 (0.32–1.90)	0.218	0.577

^a ^All participants at baseline. ^b ^Participants who at baseline reported no sick leave due to neck, shoulder and/or back disorders during the preceding 12 months and who also answered the questionnaire at the 18-month follow-up. Minimum and maximum values are presented within square brackets. ^c ^Best possible value. ^d ^Worst possible value. CI = 95% confidence interval. Odds Ratios are adjusted for age and intervention

**Table 5:** Significant predictors of future sick leave due to any cause (*Study group I*)^a ^and due to neck, shoulder and/or back disorders (*Study group II*)^b ^for factors related to health, work and leisure time respectively. Multiple regression analyses for each model.

	***Study group I^a^***	***Study group II^b^***	***Study group I***	***Study group II***
***Models***	***(n = 443) OR *** ***(95%CI)***	***(n = 274) OR *** ***(95%CI)***	***P value***	***P value***
Predictors related to health				
- Low back disorders during the year before baseline	1.89 (1.20–2.97)	4.45 (1.27–15.77)	0.006	0.007
- Any sick leave during the year before baseline 0 days/≥1 days	6.40 (3.97–10.31)		<10^-3^	
- Sick leave during the year before baseline (other reasons than disorders of the neck, shoulder and/or back) < 9 days/≥9 days		3.30 (1.33–8.17)		0.011
Predictors related to work				
- High strain work	-	2.34 (1.05–5.23)	-	0.041
Predictors related to leisure time				
- High perceived physical exertion in domestic work	-	2.56 (1.12–5.86)	-	0.026

^a ^All participants at baseline. ^b ^Participants who at baseline reported no sick leave due to neck, shoulder and/or back disorders during the preceding 12 months and who also answered the questionnaire at the 18-month follow-up. OR = Odds Ratio. CI = 95% confidence interval. Odds Ratios are adjusted for age and intervention
